# Low expression levels of microRNA-124-5p correlated with poor prognosis in colorectal cancer via targeting of SMC4

**DOI:** 10.1002/cam4.309

**Published:** 2014-08-01

**Authors:** Takafumi Jinushi, Yoshihiko Shibayama, Ichiro Kinoshita, Satoshi Oizumi, Masahisa Jinushi, Tadahiro Aota, Toshiyuki Takahashi, Shoichi Horita, Hirotoshi Dosaka-Akita, Ken Iseki

**Affiliations:** 1 Graduate School of Pharmaceutical Sciences, Clinical Pharmaceutics and Therapeutics, Hokkaido UniversitySapporo, Japan; 2 Hokkaido Gastroenterology HospitalSapporo, Japan; 3 Graduate School of Pharmaceutical Sciences, Education Research Center for Clinical Pharmacy, Hokkaido UniversitySapporo, Japan; 4 Graduate School of Medicine, Department of Medical Oncology, Hokkaido UniversitySapporo, Japan; 5 Graduate School of Medicine, First Department of Medicine, Hokkaido UniversitySapporo, Japan; 6 Research Center for Infection-Associated Cancer, Institute for Genetic MedicineSapporo, Japan

**Keywords:** Colorectal cancer, EZH2, MFGE8, miR-124-5p, miR-26a, SMC4

## Abstract

A component of polycomb repressor complex 2, enhancer of zeste homolog 2 (EZH2), plays an important role in tumor malignancy and metastasis, while milk fat globule-epidermal growth factor-factor 8 (MFGE8) plays a key role in tumor progression and prognosis. MicroRNAs (miRs) are also critically involved in various physiological and pathological processes. We here evaluated the relationship between overall survival (OS) in colorectal cancer patients and the expression of onco-miRs and miRs, which may target *EZH2* and *MFGE8*. Plasma and formalin-fixed paraffin-embedded (FFPE) samples were obtained from 71 colorectal cancer patients. The expression levels of miRs complementary to *EZH2* and *MFGE8* mRNA and cancer malignancies were evaluated. The miRs analyzed were as follows: miR-16, miR-21, miR-26a, miR-34a, miR-98, miR-101-3p, miR-101-5p, miR-124-5p (also known as miR-124*), miR-126-3p, miR-126-5p, miR-210, miR-217, and miR-630. The plasma expression levels of *MFGE8* in completely resected patients were significantly lower than those in unresectable patients. Lower miR-26a expression levels were correlated with a higher probability of OS. Higher miR-124-5p expression levels in plasma and FFPE samples were correlated with a higher probability of OS. The transfection of mimic miR-124-5p into WiDr and COLO201 cells inhibited the expression of structural maintenance of chromosomes 4 (*SMC4*) mRNA. Our results indicate that miR-124-5p may target the tumorigenesis gene, SMC4, which suggests that expression levels of miR-124-5p in plasma and FFPE samples; therefore, the expression of *MFGE8*, miR-26a, and miR-124-5p in plasma may be used as biomarkers to determine the prognosis of colorectal cancer patients.

## Introduction

Colorectal cancer remains a significant cause of mortality worldwide. Despite an earlier diagnosis and advances in available treatments, many colorectal cancers remain incurable [1, 2]. Molecular profiling will assist in the development of personalized treatment strategies [3]. Enhancer of zeste homolog 2 (EZH2), a component of the oncogene polycomb repressive complex 2, exhibits histone methyltransferase activity and induces the methylation of lysine residues in histone H3. EZH2 was previously shown to be overexpressed in cancers, and EZH2 expression levels correlated with aggressiveness, metastasis, and a poor prognosis [4, 5]. Milk fat globule-epidermal growth factor-factor 8 (MFGE8) plays an important role in controlling the progression of various inflammatory diseases. It is also involved in tumor progression and prognosis [6, 7].

MicroRNAs (miRs) have been shown to negatively regulate gene expression by binding to complementary sequence sites in the 3′-untranslated regions of the mRNAs of protein-coding genes, thereby degrading or blocking the translation of these mRNAs. MicroRNAs are known to play an important role in various physiological and pathological processes, such as apoptosis, cell proliferation, and differentiation, which indicates their functionality in carcinogenesis as tumor suppressor genes or oncogenes [8]. MicroRNAs have recently been detected in body fluids, such as serum, plasma, and saliva. Although initially considered to be unstable RNA molecules, circulating miRs are now known to be highly stable and readily detected in plasma. Exosomes are microvesicles with an endocytic origin that are released from various cells into the extracellular space. Exosomes have been detected in cell culture supernatants as well as body fluids, and are composed of a lipid bilayer. They contain mRNAs and miRs, which are enclosed in side exosomes and are secreted into the extracellular space [9, 10].

*EZH2* was previously shown to be suppressed by miR-101-3p (also known as miR-101) and miR-26a [11–14]. A bioinformatics, MicroRNA.org (http://www.microrna.org) is a comprehensive resource of microRNA target predictions and expression profiles. Target predictions are based on a development of the miRanda algorithm which incorporates current biological knowledge on target rules and on the use of an up-to-date compendium of mammalian microRNAs [15]. The microRNA.org predicted that *EZH2* may be targeted by miR-26a, miR-34a, miR-98, miR-101-3p, miR-217, and miR-630. Previous studies suggested that miR-16, miR-21, miR-34a, miR-101-3p, miR-124-5p (also known as miR-124*), miR-126-3p (also known as miR-126), miR-126-5p (also known as miR-126*), miR-210, miR-217, and miR-630 may be used as prognostic and diagnostic biomarkers for cancer [16–22]. MicroRNA.org also predicted that miR-124-5p may target structural maintenance of chromosomes 4 (*SMC4*). SMC4 is a core subunit of condensin I and II, which are large protein complexes, is involved in chromosome condensation, and has been associated with tumorigenesis [23]. However, the inhibitory effects of miR-124-5p on the expression of *SMC4* mRNA have not yet been elucidated in detail.

The relationship between overall survival (OS) in colorectal cancer patients and the expression of miRs, which may target *EZH2* and *MFGE8* and have been linked to cancer, was examined using plasma and FFPE samples. To investigate the involvement in survival benefit of miR-124-5p, the inhibitory effects of a possible target mRNA of miR-124-5p, *SMC4*, were evaluated.

## Materials and Methods

### Reagents

The QuantiTect Primer Assay, miScript Primer Assay, miScript Reverse Transcription Kit, and synthetic microRNA mimic and miScript SYBR Green PCR Kit were purchased from Qiagen (Valencia, CA). The real-time PCR master mix THUNDERBIRD SYBR qPCR Mix and reverse transcriptase (RT), ReverTra Ace was purchased from TOYOBO Co., Ltd. (Osaka, Japan). The High Pure RNA Isolation Kit and High Pure RNA Paraffin Kit were purchased from Roche Diagnostics GmbH (Mannheim, Germany). Dulbecco's Modified Eagle's Medium (DMEM) was purchased from Sigma-Aldrich (St. Louis, MO). The human colon adenocarcinoma cell lines, WiDr (JCRB0224) and COLO201 (JCRB0226) were purchased from the Japanese Collection of Research Bioresources (JCRB) Cell Bank (Osaka, Japan). These cell lines were tested and authenticated by the JCRB Cell Bank. Lipofectamine 2000 reagent was purchased from Life technologies (Carlsbad, CA). Synthetic SMC4 siRNA (sense: gcccaagaauguguaaacu, anti: aguuuacacauucuugggc) [23] was obtained from Bioneer Corporation (Daejeon, Republic of Korea). The PCR primers for SMC4 (sense: gagaaaattctgggaccttt, anti: tctgaatgtccttgtgttca) and glyceraldehyde-3-phosphate dehydrogenase (GAPDH, sense: aacagcctcaagatcatcagc, anti: ggatgatgttctggagagcc) [23] were obtained from Hokkaido System Science Co., Ltd. (Sapporo, Japan). All other reagents were purchased from Wako Pure Chemical Industries (Osaka, Japan).

### Patients and sample collection

We examined 71 patients with colorectal cancer who were recruited at Hokkaido Gastroenterology Hospital. All patients received chemotherapy according to the Japanese Society for Cancer of the Colon and Rectum Guidelines [24]. This study was approved by the Ethical Committee at the affiliations. Written informed consent was obtained from all patients. The study protocol was approved by the Institutional Review Board and conformed to the guidelines of the 2008 Declaration of Helsinki. Blood samples for medical testing purposes were collected into ethylenediaminetetraacetic acid (EDTA) tubes. Plasma was separated from the residuum of the samples for the blood cell count inspection prior to chemotherapy. Plasma samples stored at −80°C. Formalin-fixed paraffin-embedded (FFPE) samples were obtained from tumor histology. The characteristics of the study population are shown in Table 1.

**Table 1:** Patient characteristics.

Characteristics	Unresectable (*n* = 49)	Completely resected (*n* = 22)	*P*
Age			
Mean (SD)	63.0 (11.4)	59.1 (6.8)	
Range	30–83	47–74	
Sex			0.22
Male	34	12	
Female	15	10	
TNM classification			<0.0001
ΙΙ	0	3	
ΙΙΙ	0	13	
IV	49	6	
Primary lesion			0.19
Colon	19	12	
Rectum	20	9	
Other	10	1	
Histology			0.07
Tubular adenocarcinoma	44	19	
Mucinous adenocarcinoma	1	3	
Other	4	0	
Chemotherapy			0.03
mFOLFOX6	14	12	
FOLFIRI	9	1	
IRIS	11	0	
XELOX	3	4	
Capecitabine	5	1	
Other	7	4	

Statistical analysis for single comparisons was performed using the two-tailed *χ*^2^ test or Fisher's exact test (expected frequency < 5). Comparisons between two groups were performed with the Mann–Whitney *U*-test. Staging was classified according the UICC TNM classification of malignant tumors. mFOLFOX6 comprised infusional 5-fluorouracil + *l*-leucovorin + oxaliplatin, FOLFIRI comprised infusional 5-fluorouracil + *l*-leucovorin + irinotecan, IRIS consisted of S-1 (an oral prodrug of 5-fluorouracil) + irinotecan, and XELOX consisted of capecitabine + oxaliplatin.

### RNA isolation and RT-PCR

Total RNA was isolated from 200 *μ*L of plasma and FFPE samples using the High Pure RNA Isolation Kit and High Pure RNA Paraffin Kit according to the manufacturer's instructions. Single-stranded cDNA was synthesized by RT using ReverTra Ace, and single-stranded cDNA for microRNA analysis was also synthesized by RT using the miScript Reverse Transcription Kit according to the manufacturer's instructions. Real-time PCR was performed using the LightCycler 480 ΙΙ System (Version 1.5; Roche Diagnostics GmbH, Mannheim, Germany) with TaqMan gene expression assays and the THUNDERBIRD qPCR Mix or miScript SYBR Green PCR Kit according to the manufacturer's instructions. Comparative real-time RT-PCR assays were performed for each sample in triplicate. The comparative quantification cycle threshold (*C*_q_) method was used to determine the relative expression levels of the target genes. *C*_q_ values were calculated with the second derivative maximum method. GAPDH and RNU6B (U6) were analyzed as a reference gene for mRNA and microRNA, respectively [25, 26]. The cycle number difference (Δ*C*_q_ = reference genes − target genes) was calculated in each replicate. Relative target gene expression values were calculated using the mean of Δ*C*_q_ from the three replicates, that is, *μ* (Δ*C*_q_) = Σ (Δ*C*_q_)/3, and expressed as 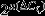 [27].

### Cell culture and transfection assays

The human colon adenocarcinoma cell lines, WiDr and COLO201 were grown in, DMEM and RPMI1640 medium, respectively, which was supplemented with 10% fetal bovine serum, 2 mmol/l-glutamine, and 100 units/mL of penicillin at 37°C in a 5% CO_2_ humidified atmosphere. The synthetic microRNA mimic or siRNA were transfected using the Lipofectamine 2000 transfection agent according to the manufacturer's protocol. In 96-well plates, 3 pmol of the mimic or siRNA was transfected into 1 × 10^5^ cells/ml using 0.4 *μ*L of Lipofectamine 2000, and cells were harvested 72 h later for RT-PCR and the 3-(4,5-dimethylthiazol-2-yl)-2,5-diphenyl tetrazolium bromide (MTT) assay [28].

### Statistical analysis

Comparisons between two groups were performed with the Mann–Whitney *U*-test. Comparisons between three groups were performed with the Tukey–Kramer test. Categorical variables were analyzed with the two-tailed *χ*^2^ test or Fisher's exact test (expected frequency < 5). Survival was plotted with Kaplan–Meier curves, taking the interval from the date of colorectal cancer to death or last contact. Comparisons between each group were performed with the log-rank test. OS and progression-free survival (PFS) were evaluated using the Cox proportional hazards model. The relationship was analyzed using univariate analysis. All indicated *P*-values are two-sided. *P < 0.05, **P < 0.01, ***P < 0.001.

## Results

### The relationship between microRNA and OS in patients with unresectable colorectal cancer

**Figure 1:**
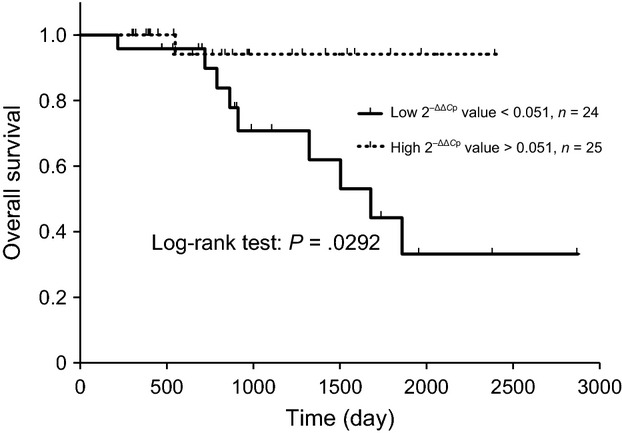
Kaplan–Meier OS curves for patients with unresectable colorectal cancer based on low and high plasma miR-124-5p expression levels. Kaplan–Meier plots showing estimates of overall survival (OS) probabilities grouped according to miR-124-5p expression levels in a completely independent set of colorectal cancer patients. The dotted line curve represents samples that expressed high levels of miR-124-5p (above median), whereas the black curve corresponds to samples that expressed low miR-124-5p levels (below median). Discontinuations of observations were indicated by spines on the lines. Comparisons between each group were performed with the log-rank test.

**Figure 2:**
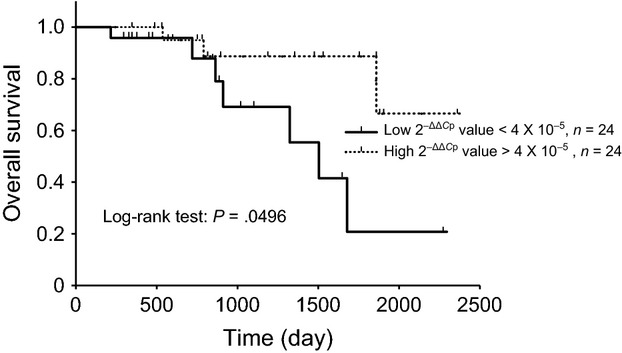
Kaplan–Meier overall survival (OS) curves for patients with unresectable colorectal cancer based on low and high miR-124-5p expression levels in formalin-fixed paraffin-embedded (FFPE) samples. FFPE samples were obtained from surgery or biopsy for histological diagnosis. Kaplan–Meier plots showing estimates of OS probabilities grouped according to miR-124-5p expression levels in a completely independent set of colorectal cancer patients. The dotted line curve represents samples that expressed high levels of miR-124-5p (above median), whereas the black line curve corresponds to samples that expressed low levels of miR-124-5p (below median). Discontinuations of observations were indicated by spines on the lines. Comparisons between each group were performed with the log-rank test.

**Figure 3:**
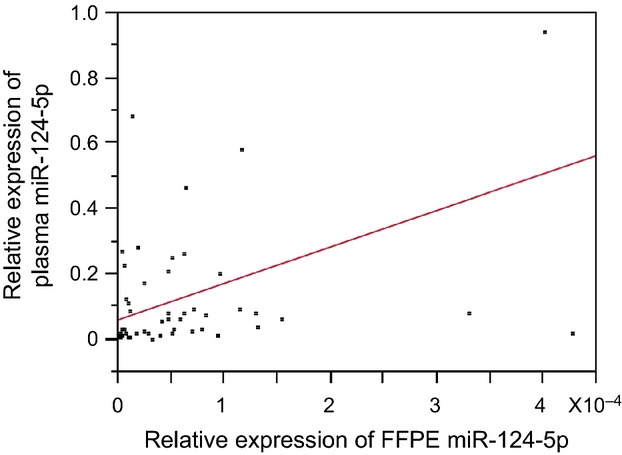
The correlation between expression levels of formalin-fixed paraffin-embedded (FFPE) miR-124-5p and plasma miR-124-5p. The relationship was analyzed by using univariate analysis, *r* = 0.451, 95% confidence interval: 0.189 − 0.654, *P* = 0.002. The plots from paired samples from the same patient and regression line were indicated.

The relationship between plasma RNA expression levels and survival duration was evaluated. Higher plasma miR-124-5p expression levels (more than the median value) were correlated with a higher probability of OS (Fig. 1). Patient characteristics are shown in Table 2. No significant difference was observed between the two groups. Expression levels of RNAs were not observed between in the two groups (data not shown). Higher FFPE miR-124-5p expression levels were also significantly correlated with a higher probability of OS (Fig. 2). Patient characteristics are shown in Table 3, and no significant differences were observed between the two groups. Plasma miR-124-5p expression levels in the high group (divided by FFPE miR-124-5p expression) were significantly higher than those in the low group (Table 4). In the present study, there was a significant correlation coefficients between plasma miR-124-5p and FFPE miR-124-5p expression levels (Fig. 3. Univariate analysis: *r* = 0.451, 95% confidence interval: 0.189 − 0.654, *P* = 0.002).

**Table 2:** Patient characteristics with grouping based on microRNA-124-5p expression levels in plasma.

Characteristics	miR-124-5p low (*n* = 24)	miR-124-5p high (*n* = 25)	*P*
Age			
Mean (SD)	62.9 (14.5)	63.0 (7.7)	0.62
Range	30–83	48–78	
Sex			0.69
Male	16	18	
Female	8	7	
Primary lesion			0.11
Colon	6	13	
Rectum	13	7	
Other	5	5	
Histology			1.00
Tubular adenocarcinoma	22	22	
Other	2	3	
Chemotherapy			0.54
mFOLFOX6	5	9	
FOLFIRI	3	6	
IRIS	6	5	
XELOX	2	1	
Capecitabine	3	2	
Other	5	2	

Statistical analysis for single comparisons was performed using the two-tailed *χ*^2^ test or Fisher's exact test (expected frequency < 5). Comparisons between two groups were performed with the Mann–Whitney *U*-test.

**Table 3:** Patient characteristics with grouping based on microRNA-124-5p expression levels in FFPE samples.

Characteristics	miR-124-5p low (*n* = 24)	miR-124-5p high (*n* = 24)	*P*
Age			
Mean (SD)	63.1 (12.3)	63.8 (9.9)	0.82
Range	30–82	40–83	
Sex			0.35
Male	18	15	
Female	6	9	
Primary lesion			0.35
Colon	10	9	
Rectum	11	8	
Other	3	7	
Histology			0.35
Tubular adenocarcinoma	23	20	
Other	1	4	
Chemotherapy			0.74
mFOLFOX6	8	5	
FOLFIRI	5	4	
IRIS	5	6	
XELOX	2	1	
Capecitabine	2	3	
Other	2	5	

Statistical analysis for single comparisons was performed using the two-tailed *χ*^2^ test or Fisher's exact test (expected frequency < 5). Comparisons between two groups were performed with the Mann–Whitney *U*-test. formalin-fixed paraffin-embedded.

**Table 4:** MicroRNA expression levels in plasma and FFPE samples, and microRNA 124-5p expression in FFPE samples.

	Plasma microRNA (×10^−3^)		FFPE microRNA (×10^−5^)	
	Low (*n* = 24)	High (*n* = 25)	Low (*n* = 24)	High (*n* = 24)
miR-16	363 ± 253	293 ± 177	94 ± 81	182 ± 153
miR-21	85 ± 131	105 ± 64	581 ± 375	801 ± 597
miR-26a	432 ± 281	388 ± 224	184 ± 120	273 ± 201
miR-34a	119 ± 272	127 ± 183	301 ± 615	267 ± 239
miR-98	36 ± 48	35 ± 43	6.4 ± 3.6	14.8 ± 20.8
miR-101-3p	58 ± 65	124 ± 138*	86 ± 83	147 ± 164
miR-101-5p	65 ± 76	91 ± 75	3.3 ± 2.6	22.4 ± 32.9***
miR-124-5p	91 ± 152	158 ± 219*	1.4 ± 1.2	11.8 ± 10.9***
miR-126-3p	122 ± 138	130 ± 124	551 ± 493	884 ± 814
miR-126-5p	207 ± 121	244 ± 167	180 ± 178	285 ± 291
miR-210	394 ± 192	415 ± 230	12 ± 5	19 ± 14
miR-217	20 ± 43	35 ± 37	0.21 ± 0.15	0.59 ± 0.52
miR-630	94 ± 78	84 ± 58	2.6 ± 1.8	5.2 ± 8.7

The two groups was divided between median value expression of miR-124-5p in FFEP, high group corresponded to above median, low group corresponded to below median. Expression levels were calculated using the 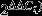 2^−ΔΔCq^ method. Δ*C*_q_ was defined as the mean *C*_q_ value for a specific RNA in an individual sample. Each value indicates the mean ± standard deviation. Statistical analysis for single comparisons was performed using the Mann–Whitney *U*-test; FFPE, formalin-fixed paraffin-embedded.

* *P* < 0.05;

*** *P* < 0.001.

**Figure 4:**
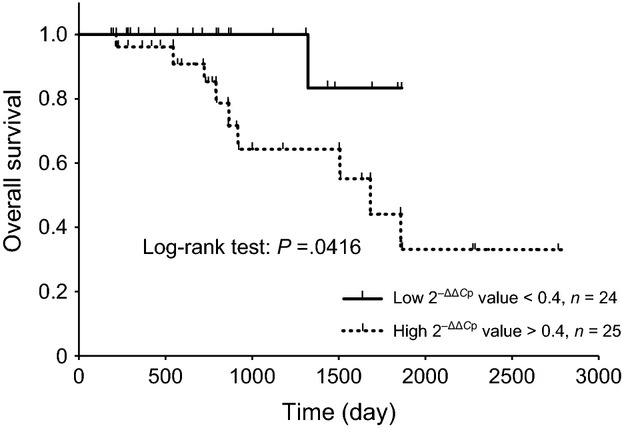
Kaplan–Meier overall survival (OS) curves for two groups defined based on low and high plasma miR-26a expression levels. Kaplan–Meier plots showing estimates of OS probabilities grouped according to miR-26a expression levels in a completely independent set of colorectal cancer patients. The dotted line curve represents samples that expressed high levels of miR-26a (above median), whereas the black line curve corresponds to samples that expressed low miR-26a levels (below median). Discontinuations of observations were indicated by spines on the lines. Comparisons between each group were performed with the log-rank test.

Lower plasma miR-26a expression levels were correlated with a higher probability of OS (Fig. 4). Cox proportional hazards models also estimated a significant lower hazard ratio in the plasma miR-124-5p higher expression group and plasma miR-26a lower expression group (Table 5). The FFPE miR-124-5p higher expression group was not correlated with a lower hazard ratio of OS (Table 5). No significant relationship was observed between the expression levels of *EZH2*, *MFGE8*, and other miRs, OS, or PFS. The expression levels of miR-26a and miR-124-5p did not correlated with PFS or the hazard ratio (Table 5).

**Table 5:** Adjusted hazard ratios of patients with colorectal cancer in the high-expression group versus the low-expression group.

	Hazard ratio (95% CI)	*P*
Plasma miR-124-5p		
OS	0.147 (0.008–0.789)	0.022
PFS	0.624 (0.291–1.300)	0.209
FFPE miR-124-5p		
OS	0.281 (0.059–1.039)	0.057
PFS	1.036 (0.490–2.252)	0.926
Plasma miR-26a		
OS	6.044 (1.097–112.5)	0.037
PFS	1.262 (0.603–2.658)	0.535

Values indicated hazard ratios and 95% confidence intervals. Statistical analysis was performed using the Cox proportional hazards model. FFPE, formalin-fixed paraffin-embedded; OS, overall survival; PFS, progression-free survival; 95% CI, 95% confidence interval.

### Differences in plasma RNA expression levels between patients with unresectable and completely resected cancer

RNA expression levels in the plasma of unresectable and completely resected patients were evaluated. Patient characteristics are shown in Table 1. *MFGE8* expression levels in completely resected patients were significantly lower than those in unresectable patients (Table 6). MiR-26a expression levels in completely resected patients were low, but were not significantly different from those in unresectable patients (*P* = 0.08; Table 6).

**Table 6:** Expression levels of RNAs in plasma samples.

	Unresectable (*n* = 49)	Completely resected (*n* = 22)		Unresectable (*n* = 49)	Completely resected (*n* = 22)
miR-16	0.317 ± 0.202	0.256 ± 0.219	miR-126-3p	0.124 ± 0.129	0.104 ± 0.198
miR-21	0.095 ± 0.101	0.064 ± 0.070	miR-126-5p	0.244 ± 0.176	0.161 ± 0.137
miR-26a	0.429 ± 0.264	0.304 ± 0.232	miR-210	0.404 ± 0.208	0.396 ± 0.288
miR-34a	0.142 ± 0.267	0.242 ± 0.417	miR-217	0.029 ± 0.042	0.036 ± 0.063
miR-98	0.037 ± 0.045	0.039 ± 0.061	miR-630	0.134 ± 0.313	0.072 ± 0.086
miR-101-3p	0.095 ± 0.113	0.140 ± 0.190	*MFGE8*	0.033 ± 0.076	0.014 ± 0.019*
miR-101-5p	0.077 ± 0.075	0.144 ± 0.213	*EZH2*	0.021 ± 0.043	0.009 ± 0.015
miR-124-5p	0.123 ± 0.188	0.117 ± 0.174			

Expression levels were calculated using the 2^−ΔΔCq^ method. Δ*C*_q_ was defined as the mean *C*_q_ value for a specific RNA in an individual sample. Each value indicates the mean ± standard deviation. Statistical analysis for single comparisons was performed using the Mann–Whitney *U*-test.

* *P* < 0.05.

### MicroRNA-124-5p-targeted SMC4 and inhibited cell growth

**Figure 5:**
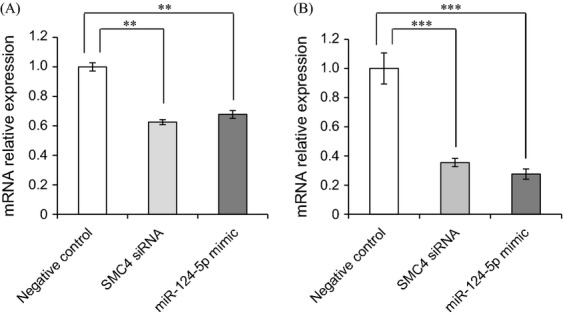
The miR-124-5p mimic inhibited *SMC4* mRNA expression in WiDr and COLO201 cells. The synthetic miR-124-5p microRNA mimic or SMC4 siRNA inhibited *SMC4* mRNA expression in WiDr (A) and COLO201 (B) cells. The synthetic microRNA mimic or siRNA were transfected using Lipofectamine 2000 transfection agent. Cells were harvested for 72 h. Values represent the relative ratio of target gene per *GAPDH*, mean ± SEM, to the control from eight independent experiments. The control group was transfected with negative control RNA (AllStars Negative Control siRNA, Qiagen). miR-124-5p: the mimic of miR-124-5p was transfected; SMC4 siRNA: synthetic SMC4 siRNA was transfected. Statistical analysis was performed using the Tukey–Kramer test; **P < 0.01, ***P < 0.001.

**Figure 6:**
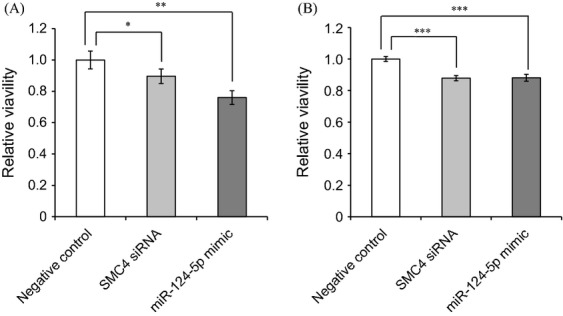
The miR-124-5p mimic inhibited cell viability in WiDr and COLO201 cells. The synthetic miR-124-5p microRNA mimic or SMC4 siRNA inhibited cell viability in WiDr (A) and COLO201 (B) cells. The synthetic microRNA mimic or siRNA were transfected using Lipofectamine 2000 transfection agent. Cells were harvested for 72 h. Cell viabilities were evaluated 3-(4,5-dimethylthiazol-2-yl)-2,5-diphenyl tetrazolium bromide (MTT) assay, which was described in detail in the method. Values represent the relative ratio of negative control treatment, mean ± SEM, to the control from eight independent experiments. The control group was transfected with negative control RNA (AllStars Negative Control siRNA, Qiagen). miR-124-5p: the mimic of miR-124-5p was transfected; SMC4 siRNA: synthetic SMC4 siRNA was transfected. Statistical analysis was performed using the Tukey–Kramer test; *P < 0.05, **P < 0.01, ***P < 0.001.

The microRNA.org predicted that miR-124-5p may target *SMC4*. Transfection of the miR-124-5p mimic or siRNA was examined. Transfection of SMC4 siRNA into WiDr and COLO201 cells significantly downregulated the expression of *SMC4* mRNA. Transfection of the miR-124-5p mimic into WiDr and COLO201 cells also significantly downregulated the expression of *SMC4* mRNA (Fig. 5). Transfection of miR-124-5p mimic or SMC4 siRNA into WiDr and COLO201 cells reduced cell viability (Fig. 6).

## Discussion

*EZH2*, *MFGE8*, and miRs expression levels in plasma and FFPE samples, and OS in colorectal cancer patients were evaluated. Previous studies reported that EZH2 is a tumorigenic gene that correlates with cancer progression and a poor prognosis [4, 29]. In the present study, a correlation was not observed between OS and *EZH2* expression levels in unresectable patients. Although miR-101 was shown to negatively regulate the expression of *EZH2*, a correlation was not observed between the expression of *EZH2* and miR-101 in plasma and FFPE samples in the present study (data not shown). Plasma *EZH2* expression levels were higher in unresectable patients than completely resected patients, but this difference was not significant (*P* = 0.4). Recent studies demonstrated that MFGE8 correlated with tumor malignancy and microenvironment [6, 30, 31]. Plasma *MFGE8* expression levels were significantly higher in unresectable patients than in completely resected patients (Table 6). Previous studies reported that the expression of *MFGE8* was significantly higher in tumors than in normal tissues. Patients with primary tumors that expressed *MFGE8* had significantly shorter survival periods than those with primary tumors that did not express *MFGE8* [32]. The results of the present study suggest that *MFGE8* mRNA released into plasma from tumors can be used as a diagnostic biomarker.

There have been some reports microRNAs are released into blood from tumor cells. Skog et al. reported that tumor-derived microvesicles which contained mRNA, microRNA, and angiogenic proteins served as a means of delivering genetic information, to recipient cells in the tumor environment [22]. In the present study, there was a significant correlation coefficient between plasma miR-124-5p and FFPE miR-124-5p expression levels (Fig. 3). However, it was not clear whether miR-124-5p in plasma was derived from tumor.

Few studies have examined the function of miR-124-5p. Anwar et al. reported that the expression of miR-124-5p (miR-124*) was significantly higher in nonmethylated hepatocellular carcinoma than in methylated samples [21]. The present study is the first to examine the relationship between the function of miR-124-5p and prognosis of patients with colorectal cancer. Wang et al. reported that the downregulation of miR-124 (miR-124-3p) correlated with a worse prognosis in patients with colorectal cancer [33]. These results confirmed the relationship between microRNA124-2, the gene of miR-124-5p, and miR-124-3p, and a worse prognosis.

The microRNA.org predicted that miR-124-5p may target *SMC4*. We examined the inhibitory effects of the miR-124-5p mimic on the expression of *SMC4* mRNA. Zhou et al. reported that the expression of *SMC4* was correlated with tumor size, de-differentiation, advanced stages, and vascular invasion of primary liver cancers, while the knockdown of *SMC4* expression reduced the proliferation of hepatocellular carcinoma cells [23]. Zhai et al. found that the knockdown of *SMC4* led to a chromosomal separation deficiency [34]. A previous study suggested that downregulation of *SMC4* may reduce cell proliferation. The knockdown of *SMC4* was shown to result in severe defects in chromosome assembly in HeLa Cells [35]. The present study demonstrated that miR-124-5p inhibited the tumorigenesis gene, *SMC4*, which upregulated the expression of miR-124-5p, thereby improving the OS of colorectal cancer patients.

The present study showed that lower miR-26a expression levels correlated with a higher probability of OS (Fig. 4). Qian et al. recently reported that miR-26a promoted tumor growth and angiogenesis in glioma [36]. The overexpression of miR-26a was shown to increase the proliferation of cholangiocarcinoma cells and colony formation in vitro [37]. These findings suggested that the upregulation of miR-26a may promote tumor growth and malignancy. The present study demonstrated that higher miR-26a expression levels were associated with a lower probability of OS, which indicated that miR-26a in plasma may be used as a biomarker to determine the prognosis of colorectal cancer patients.

In conclusion, the results of the present study demonstrated that *MFGE8* expression levels were significantly lower in completely resected patients than in unresectable patients. Furthermore, higher miR-26a expression levels, and lower miR-124-5p expression levels were associated with a lower probability of OS; therefore the expression of *MFGE8*, miR-26a and miR-124-5p in plasma may be used as biomarkers to determine the prognosis of colorectal cancer patients.
